# Pulmonary enteric adenocarcinoma with progression disease after second - line therapy: a case report

**DOI:** 10.3389/fonc.2025.1509026

**Published:** 2025-01-23

**Authors:** Ya Guo, Bin Zhang, Heng Zhang, Yunbin Gao, Haibo Zhao, Pei Jiang, Qing-Qing Yu

**Affiliations:** ^1^ Department of Oncology, Jining No.1 People’s Hospital, Jining, China; ^2^ Radiology Department, Jining No.1 People’s Hospital, Jining, China; ^3^ Translational Pharmaceutical Laboratory, Jining No.1 People’s Hospital, Jining, China

**Keywords:** pulmonary enteric adenocarcinoma, non-small cell lung cancer, diagnosis, treatment, prognosis

## Abstract

Pulmonary enteric adenocarcinoma (PEAC, also known as Enteric-type adenocarcinoma of the lung, lung - ETAC) is a rare subtype of non-small cell lung cancer (NSCLC) that has the same morphological and immunohistochemical characteristics as colorectal adenocarcinoma and requires gastroenteroscopy to rule out lesions of enteric origin. As a rare solid tumor in lung cancer, PEAC has unique clinical outcome, imaging, pathological and molecular characteristics, and poor prognosis. However, the molecular characteristics and therapeutic biomarkers of PEAC are unclear, and its treatment remains challenging. In this case, we describe a 61-year-old man diagnosed with advanced primary PEAC with KRAS mutation. In the case of unknown PD-L1 expression status, first-line treatment was given to lung adenocarcinoma regimen (immunotherapy combined with chemotherapy), progression occurred after 2 cycles, and progression-free survival (PFS) was 1.5 months. Then the second-line XELOX regimen (oxaliplatin combined with capecitabine) was adjusted. The lesions were significantly reduced after 2 and 4 cycles, and the disease progressed again after 6 cycles, with a PFS of 4.5 months. Anlotinib targeted drugs were selected for third-line treatment, but considering the overall poor condition of the patient, the patient himself refused further treatment. Finally, after discharge, the patient went to the local hospital for nutritional support and symptomatic treatment. The results suggest that standard first-line therapies (immunotherapy plus chemotherapy) and colorectal cancer regimens may have a relatively limited impact on survival in KRAS-driver positive advanced PEAC.

## Introduction

Lung adenocarcinoma is the most common histological type of non-small cell lung cancer (NSCLC) (1), with a variety of histopathological subtypes, among which pulmonary enteric adenocarcinoma (PEAC) is a rare and special subtype, originating in lung tissue, but its pathological features showed enteric origin or enteric morphology which was similar to lung metastatic colorectal cancer (ImCRC) (2–4). The new diagnostic criteria include colorectal morphologic cells that exceed 50% of the tumor tissue and are positive for at least one colorectal immunophenotype, including mainly tail homologous box (CDX-2), cytokeratin 20 (CK20), specific AT-sequence binding protein 2 (SATB2), and mucin 2 (MUC2), Villin, clinically excluding colorectal cancer (5, 6). PEAC was first reported in 1991 by Tsao and Fraser (7) in a case report. In 2011, PEAC was officially classified as invasive NSCLC for the first time in the International Multidisciplinary Classification of Lung Cancer jointly released by the International Association for the Study of Lung Cancer/American Thoracic Society/European Respiratory Society (IASLC/ATS/ERS) (3). It was not until 2015 that the World Health Organization (WHO) proposed diagnostic criteria, and in 2021 the WHO updated the classification again to further define the immunohistochemical criteria for the diagnosis of PEAC (8, 9). At present, there is no clear standard treatment plan for stage IV PEAC. The first-line treatment of stage IV NSCLC with KRAS G12C mutation should be similar to that of NSCLC without driver genes. Very few reports to date have described the effects of second-line treatment of patients with primary PEAC using a colorectal cancer regiment. This case describes a patient with advanced PEAC who, after rapid progression with a first-line immune plus chemotherapy regimen, experienced transient disease control with oxaliplatin plus capecitabine as a second-line treatment and subsequently relapsed again.

## Case presentation

The patient was a 61-year-old male with a smoking history of 20 cigarettes per day for 30 years. He was previously healthy, with no family history of genetic diseases, alcohol consumption, or psychiatric disorders. He went to a superior hospital in Jul 2023 due to “left abdominal pain for 1 month”, for which no prior treatment had been received. In Aug 2023, positron emission tomography-computed tomography (PET-CT) showed soft tissue lesions in the left upper lobe of the lung, FDG metabolism was increased, and lung cancer was possible. The left hilar lymph node was enlarged. The soft tissue density shadow of bilateral adrenal gland and the soft tissue density shadow of left kidney posteromedial were considered as metastases. Computed tomography (CT) -guided biopsy of the left lung lesion was subsequently performed. The pathological results suggested adenocarcinoma, but the expression of pulmonary markers was poor. Immunohistochemistry (IHC): TTF-1 (-), CK7 (+), Napsin A (-), CEA (+), CK5/6 (+), Syn (-), P63 (-), Ki-67+ (50%). Genetic test results: the mutation abundance of KRAS was 31.22%, and no EGFR, AKL, ROS-1, MET, HER-2 mutations were found. PD-L1 expression status was unknown. As the patient had multiple metastases at presentation and was clinically stage IV, radical surgery was no longer possible. The clinical stage was stage IV. The patient received two cycles of immune checkpoint inhibitor (ICI) plus platinum-based chemotherapy on Aug 19, 2023 and Sep 10, 2023, respectively, according to the following regimen: Tislelizumab 200mg d1+ pemetrexed 500mg/m2 d1+ cisplatin 75mg/m2 d2, every 3 weeks.

**Figure 1:**
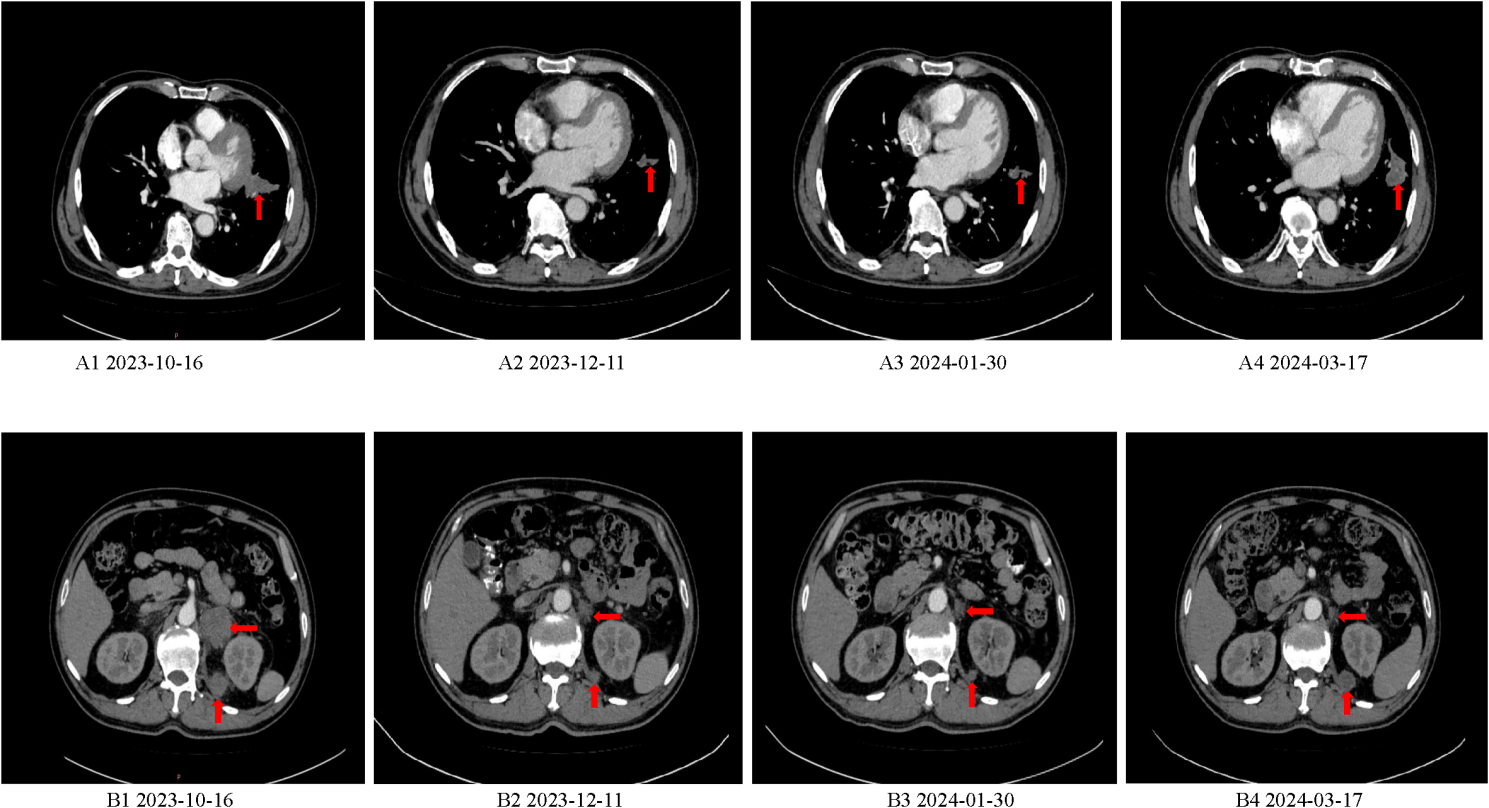
Baseline CT and CT after efficacy evaluation. **(A1, B1)** CT scan showed irregular strip soft tissue mass in the left hilar and upper lobe of the left lung, with a maximum cross-section of 4.7×3.0cm, left adrenal and left retrorenal masses and abdominal lymph nodes were considered to have metastasis. **(A2, B2)** CT scan showed that the soft tissue mass of left hilar and upper left lobe was reduced, and the metastatic lesions of left adrenal gland and posterior left kidney were smaller than before. **(A3, B3)** CT scan showed that the lesions of the left hilar - upper lobe of the left lung, double adrenal gland and internal posterior metastatic lesions of the left kidney continued to shrink compared with Dec 11, 2023. **(A4, B4)** CT scan showed that the soft tissue lesions in the left upper lobe of the lung and the metastatic lesions of posterior left kidney were enlarged again.

The patient first visited the Oncology Department of Jining First People’s Hospital on Oct 15, 2023 due to “worsening left abdominal pain for 1 week”. Due to pain affecting sleep and activity, the patient took oxycodone sustained-release tablet 10mg every 12 hours to relieve pain treatment. On Oct 16, 2023, the serum carcinoembryonic antigen (CEA) level was > 979.0ng/ml (reference range 0-5), and the carbohydrate antigen 19-9 (CA19-9) level was 280.0U/ml (reference range 0-25). Other blood routine, liver and kidney function, blood glucose and lipids, thyroid function, hepatitis B virus, electrocardiogram results were basically normal. On Oct 18, 2023, CT scan showed irregular strip soft tissue mass in the left hilar and upper lobe of the left lung, with a maximum cross-section of 4.7×3.0cm, left adrenal and left retrorenal masses and abdominal lymph nodes were considered to have metastasis (**Figures 1A1, B1**).

**Figure 2:**
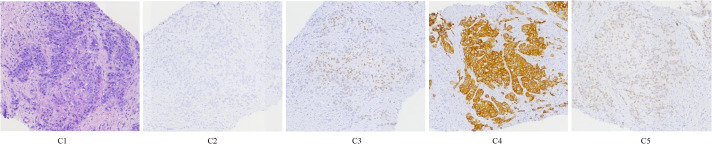
HE staining and immunohistochemistry.

After multidisciplinary discussion in our hospital, puncture biopsy was performed on the posterior medial mass of the left kidney, and the puncture histopathological results confirmed malignant tumor. Histologically, the poorly differentiated tumor cells were arranged in nested clusters, and the cells were obviously atypical (**Figure 2C1**). IHC results were as follows: CK5/6 (-), CK7 (+), SATB2 (partial +), Napsin A (-), P63 (-), TTF-1 (-), CK8/18 (+), CK20 (-), CDX-2 (+), NUT (-) (**Figures 2C2–C5**). There were no significant abnormalities in cerebral MRI, painless gastroscopy and colonoscopy and pulmonary enteric adenocarcinoma was diagnosed.

The patient began receiving 6 cycles of XELOX regimen on Oct 27, 2023, according to the following dosing schedule: oxaliplatin 240mg d1+ capecitabine 1.5g bid orally for 14 days, every 3 weeks. The left abdominal pain gradually decreased after 1 cycle of chemotherapy, and oxycodone sustained-release tablets were discontinued after 2 cycles of chemotherapy.

On Dec 11, 2023 (after 2 cycles) serum CEA levels dropped to 173.0ng/ml (reference range 0-5), CA19-9 levels dropped to 90.60U/ml (reference range 0-25). CT scan showed that the soft tissue mass of left hilar and upper left lobe was reduced, and the metastatic lesions of left adrenal gland and posterior left kidney were smaller than before, assessed the efficacy as partial remission (PR) (**Figures 1A2, B2**).

Jan 30, 2024 (after 4 cycles) blood CEA 20.60ng/ml (reference range 0-5), CA19-9 74.50U/ml (reference range 0-25). Compared with the CT scan on Dec 11, 2023, the left hilum and left upper lobe lung lesions, adrenal gland and left kidney internal posterior metastatic lesions continued to shrink, and the efficacy was assessed as continuous PR (**Figures 1A3, B3**). Mar 17, 2024 (after 6 cycles) blood CEA increased to 86.60ng/ml (reference range 0-5), CA19-9 increased to 108.50U/ml (reference range 0-25). CT scan showed that the lesions of upper lobe of the left lung and left kidney internal posterior metastatic lesions were enlarged again, and the curative effect was progression of disease (PD) (**Figures 1A4, B4**).

**Figure 3:**
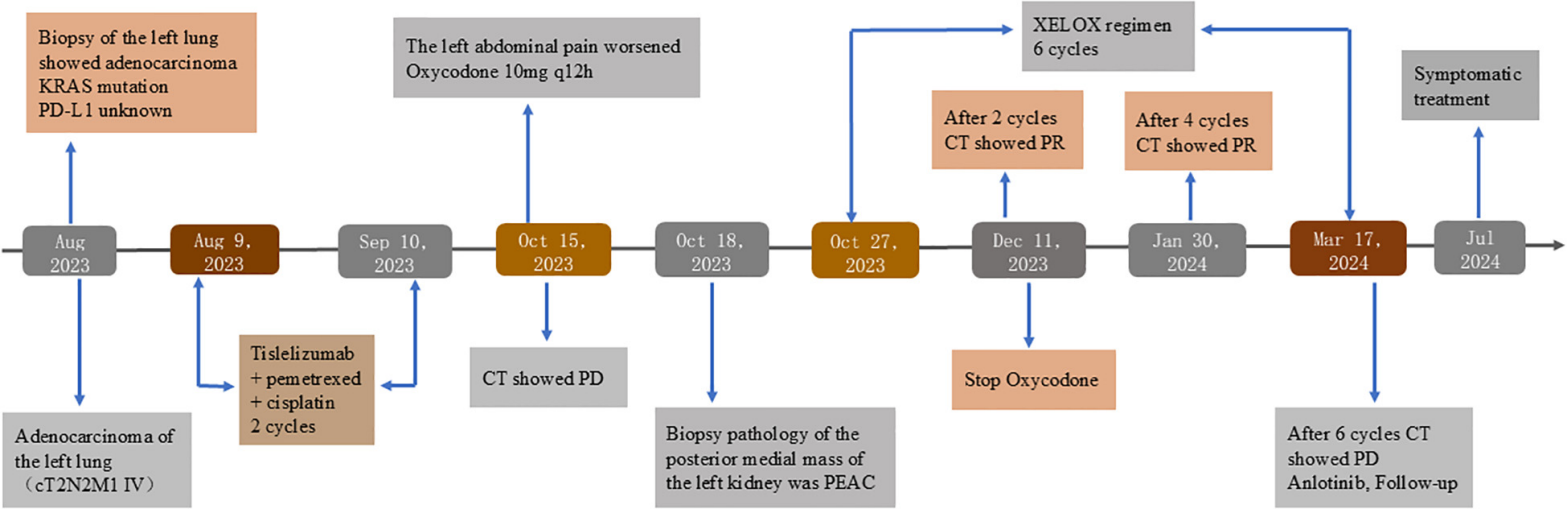
Timeline figure of the clinical course.

Although the patient selected the third-line treatment of anlotinib, the patient and family refused further treatment because the patient was less likely to benefit from this regimen than from the first - and second-line regiments. Patients were followed up until Jul 2024 and received nutritional support and symptomatic treatment at local hospitals, but their overall condition was poor. A detailed timeline figure of the clinical course is shown in **Figure 3**.

## Discussion

PEAC is a rare pathological subtype of lung adenocarcinoma, the combination of clinical manifestations, laboratory tests, histopathology, IHC, and molecular features is necessary for a definitive diagnosis of PEAC (10). Most patients with PEAC have pulmonary symptoms as the initial clinical manifestations, such as fever, fatigue, cough, sputum, chest pain, hemoptysis, chest and back pain, etc (2, 11). Serum CEA and CA19-9 are significantly elevated in PEAC. In addition, sustained high levels of CEA and CA19-9 are closely related to the pathological diagnosis and progression of PEAC (12, 13). Combined with previous PEAC-related case reports and 1 patient in our hospital, there is still no conclusion on the location, size and shape of the lesions shown by CT, and more case data are needed to summarize the related imaging findings of PEAC (12, 14–16). The histological features and immunophenotypes of PEAC are similar to those of metastatic colorectal cancer. Its diagnosis requires gastroscopy and colonoscopy to rule out primary gastrointestinal neoplasms. Specifically, more than 50% of tumors showed enteric cell morphology with components of conventionally-aggressive non-mucinous adenocarcinomas, and at least one lung and one colorectal biomarker had to be expressed simultaneously (17). In the studies related to PEAC, the immunohistochemical expressions of TTF-1 and Napsin A were inconsistent, and at least one enteric differentiation marker (including mainly CDX-2, CK20, MUC2, Villin, SATB2) was expressed, which was crucial for the diagnosis of PEAC (5, 18). In recent years, researchers have conducted more research and exploration on the precision treatment of PEAC. Liu Y et al. (19) found that KRAS mutation was the most common mutant gene in PEAC, with an incidence rate (47.1%), and the incidence of G12D and G12V was higher than that of G12A and G12C. Nottegar et al. (18) showed that the mutation frequency of KRAS was as high as 60.9% (28/46). Secondly, the mutation frequency of human epidermal growth factor receptor-2 (HER2) in PEAC is 44.4%, which is significantly higher than that in NSCLC, suggesting that anti-HER2 therapy may have a potential therapeutic effect. In contrast, EGFR, NRAS, BRAF mutations, RET fusions, and EML4-ALK rearrangements were uncommon. Nottegar et al. (18) evaluated eight patients and found no mutations in NRAS, BRAF, or EGFR. Among 127 patients analyzed by Fassi et al. (10), ROS1 (15%), RET (13%), BRAF (11%), EGFR (8%), and ALK (6%) were significantly less mutated than KRAS (31%).

At present, the treatment strategy of PEAC is similar to that of NSCLC (20). According to different clinical stages, a comprehensive treatment plan consisting mainly of surgical resection, supplemented by chemotherapy, radiotherapy, immunotherapy and targeted therapy is adopted (21, 22). In the vast majority (>90%) of patients with early stage disease, surgical treatment is the preferred approach, and improve the prognosis of patients with PEAC (10). However, there was no surgical opportunity for the patient in this article, who received advanced first-line treatment with the lung adenocarcinoma regimen (pemetrexed combined with cisplatin) and developed disease progression after 2 cycles. This was followed by a second-line regimen (oxaliplatin combined with capecitabine), and a detailed evaluation showed that the second-line regimen produced a limited therapeutic response, with a sustained decrease in blood CEA and CA99 levels, and CT showed improvement, but the disease worsened again after 6 cycles. Considering the heterogeneity of the case study and tumor treatment, no definitive conclusions can be drawn about the efficacy of the two different treatment regimens. For patients with advanced PEAC, median survival is 14 months with systemic chemotherapy only, which appears to be superior to patients with lung adenocarcinoma (23). Some studies (24, 25) have shown that systemic therapy is still controversial for patients with advanced PEAC that cannot be resectable. One option is to treat lung adenocarcinoma with platinum combined with taxol; Another option is a colorectal cancer regimen, such as oxaliplatin, irinotecan, and fluorouracil. Lin et al. (26) described a stage IV patient with disease progression after 3 cycles of first-line XELOX (oxaliplatin plus capecitabine) regimen.

For patients with stage IV PEAC, chemotherapy alone has relatively limited impact on long-term prognosis, so combination immunotherapy may be a potential direction of exploration. Liu, Yuan et al. (19) study have shown that patients with PD-L1 expression exhibit a higher degree of immune infiltration and may benefit from immunotherapy, suggesting that PD-L1 may serve as a biomarker for immunotherapy in PEAC patients. Another study showed that pembrolizumab combined with pemetrexed plus carboplatin chemotherapy effectively controlled the disease, and patients continued to reduce the lesion 5 months after stopping treatment due to adverse events, despite KRAS G12D mutation positive and PD-L1 tumor proportion score (TPS) of less than 1% (27). In comparison, Hu et al. (28) reported a case of a KRAS mutated stage IV PEAC patient receiving paclitaxel plus carboplatin combined with sintilimab immunotherapy with rapid disease progression after one cycle, similar to the results of this study. The results suggest that immunotherapy combined with chemotherapy may not be effective for KRAS-mutated PEAC. KRAS mutation as a driver gene of advanced NSCLC, the data reported in the literature are not clear, and there is significant tumor heterogeneity, indicating that KRAS dependence is the main driver of poor prognosis (29–31)

In addition to immunotherapy, the rapid research progress of targeted therapy is also a noteworthy aspect. In this case report, the patient only underwent immunohistochemistry and routine target mutation testing, without large-sample genetic testing, and more genetic mutations were unclear. The patient reported in this case had disease progression after immunotherapy combined with chemotherapy, and the addition of KRAS-targeted drugs should be considered in the later line of treatment.

Due to the rarity of the disease, little is known about the clinical prognosis of people with PEAC. PEAC is a highly heterogeneous, aggressive tumor that can metastasize rapidly. Therefore, the choice of medication regimen is very important for prognosis (16, 19). At present, there is no clear standard treatment strategy. For PEAC patients with positive driver genes, selecting appropriate molecular targeted therapy on the basis of accurately distinguishing mutant genotypes is expected to be an effective treatment plan.

## Conclusions

We report a rare case of primary advanced KRAS mutated NSCLC. Immune checkpoint inhibitors and conventional chemotherapy drugs are not sensitive to treatment, effective biomarkers and pathogenesis need to be further explored from the genomic and molecular levels, and more basic and clinical research is needed to find the best chemotherapy regimen and possibly effective targeted or immune drugs. This study promotes the further understanding of this rare non-small cell lung cancer and provides new ideas for clinical treatment options. In the future, the clinical application of immunotherapy or targeted therapy in PEAC should be further explored to improve the prognosis of patients.

## Data Availability

The original contributions presented in the study are included in the article/supplementary material. Further inquiries can be directed to the corresponding authors.

## References

[1] Wang A Han C Zhao H Zheng Z Ye X Shan R. Progress in the knowledge on the transformation of lung adenocarcinoma to small-cell lung cancer. J Cancer Res Ther. (2023) 19:14–9. doi: 10.4103/jcrt.jcrt_1842_22 37006037

[2] Bian T Zhao J Feng J Zhang Q Qian L Liu J. Combination of cadherin-17 and SATB homeobox 2 serves as potential optimal makers for the differential diagnosis of pulmonary enteric adenocarcinoma and metastatic colorectal adenocarcinoma. Oncotarget. (2017) 8:63442–52. doi: 10.18632/oncotarget.18828 PMC5609935 28969003

[3] Travis WD Brambilla E Noguchi M Nicholson AG Geisinger KR. International association for the study of lung cancer/American thoracic society/European respiratory society international multidisciplinary classification of lung adenocarcinoma. J Thorac Oncol. (2011) 6:244–85. doi: 10.1097/JTO.0b013e318206a221 PMC4513953 21252716

[4] Chen H Carrot-Zhang J Zhao Y Hu H Freeman SS Yu S. Genomic and immune profiling of pre-invasive lung adenocarcinoma. Nat Commun. (2019) 10:5472. doi: 10.1038/s41467-019-13460-3 31784532 PMC6884501

[5] Kishikawa S Hayashi T Takamochi K Ura A Sasahara N Saito T. Frequent nuclear β-catenin expression in pulmonary enteric-type adenocarcinoma according to the current World Health Organization criteria. Virchows Arch. (2023) 483:699–703. doi: 10.1007/s00428-023-03657-9 37740071

[6] Truini A Santos Pereira P Cavazza A Spagnolo P Nosseir S Longo L. Classification of different patterns of pulmonary adenocarcinomas. Expert Rev Respir Med. (2015) 9:571–86. doi: 10.1586/17476348.2015.1083428 26313326

[7] Ming-Sound Tsao RSF. Primary pulmonary adenocarcinoma with en teric differentiation. Cancer. (1991) 68:1754–7. doi: 10.1002/1097-0142(19911015)68:8<1754::aid-cncr2820680818>3.0.co;2-e 1913519

[8] Travis WD Brambilla E Nicholson AG Yatabe Y Austin JHM Beasley MB. The 2015 world health organization classification of lung tumors. J Thorac Oncol. (2015) 10:1243–60. doi: 10.1097/JTO.0000000000000630 26291008

[9] Nicholson AG Tsao MS Beasley MB Borczuk AC Brambilla E Cooper WA. The 2021 WHO classification of lung tumors: impact of advances since 2015. J Thorac Oncol. (2022) 17:362–87. doi: 10.1016/j.jtho.2021.11.003 34808341

[10] Fassi E Mandruzzato M Zamparini M Bianchi S Petrelli F Baggi A. Clinical presentation and outcome of patients with enteric-type adenocarcinoma of the lung: A pooled analysis of published cases. Lung Cancer. (2023) 179:107176. doi: 10.1016/j.lungcan.2023.107176 37015149

[11] Wang Q Zhang L Li H Liu L Sun X Liu H. Clinical features and prognosis of pulmonary enteric adenocarcinoma: A retrospective study in China and the SEER database. Front Oncol. (2023) 13:1099117. doi: 10.3389/fonc.2023.1099117 37051525 PMC10083384

[12] Zhao L Huang S Liu J Zhao J Li Q Wang HQ. Clinicopathological, radiographic, and oncogenic features of primary pulmonary enteric adenocarcinoma in comparison with invasive adenocarcinoma in resection specimens. Medicine. (2017) 96:e8153. doi: 10.1097/MD.0000000000008153 28953659 PMC5626302

[13] Chen M Liu P Yan F Xu S Jiang Q Pan J. Distinctive features of immunostaining and mutational load in primary pulmonary enteric adenocarcinoma: implications for differential diagnosis and immunotherapy. J Transl Med. (2018) 16:81. doi: 10.1186/s12967-018-1449-z 29587865 PMC5870381

[14] Wang CX Liu B Wang YF Zhang RS Yu B Lu ZF. Pulmonary enteric adenocarcinoma: a study of the clinicopathologic and molecular status of nine cases. Int J Clin Exp Pathol. (2014) 7:1266–74. PMC3971340 24696747

[15] Tu LF Sheng LY Zhou JY Wang XF Wang YH Shen Q. Diagnosis and treatment of primary pulmonary enteric adenocarcinoma: Report of Six cases. World J Clin cases. (2021) 9:9236–43. doi: 10.12998/wjcc.v9.i30.9236 PMC8567515 34786410

[16] Li H Cao W. Pulmonary enteric adenocarcinoma: a literature review. J Thorac Dis. (2020) 12:3217–26. doi: 10.21037/jtd-19-4171 PMC7330779 32642243

[17] Thway K Nicholson AG Lawson K. Primary pulmonary myxoid sarcoma with EWSR1-CREB1 fusion: A new tumor entity. Am J Surg Pathol. (2011) 35:1722–32. doi: 10.1097/PAS.0b013e318227e4d2 21997693

[18] Nottegar A Tabbò F Luchini C Brunelli M Bria E Veronese N. Pulmonary adenocarcinoma with enteric differentiation: immunohistochemistry and molecular morphology. Appl Immunohistochem Mol Morphol. (2018) 26:383–7. doi: 10.1097/PAI.0000000000000440 27753661

[19] Liu Y Lu T Yuan M Chen R Lu J Wang H. Genomic and transcriptomic insights into the precision treatment of pulmonary enteric adenocarcinoma. Lung Cancer. (2023) 179:107169. doi: 10.1016/j.lungcan.2023.03.005 37003209

[20] Zuo Y Bai H Ying JM Wang J. Progress in pulmonary enteric adenocarcinoma. Zhonghua Zhong Liu Za Zhi. (2022) 44:321–5. doi: 10.3760/cma.j.cn112152-20200818-00753 35448919

[21] Alduais Y Zhang H Fan F Chen J Chen B. Non-small cell lung cancer (NSCLC): A review of risk factors, diagnosis, and treatment. Med (Baltimore). (2023) 102:e32899. doi: 10.1097/MD.0000000000032899 PMC11309591 36827002

[22] Duma N Santana-Davila R Molina JR. Non-small cell lung cancer: epidemiology, screening, diagnosis, and treatment. Mayo Clin Proc. (2019) 94:1623–40. doi: 10.1016/j.mayocp.2019.01.013 31378236

[23] Treat J Scagliotti GV Peng G Monberg MJ Obasaju CK Socinski MA. Comparison of pemetrexed plus cisplatin with other first-line doublets in advanced non-small cell lung cancer (NSCLC): A combined analysis of three phase 3 trials. Lung Cancer. (2012) 76:222–7. doi: 10.1016/j.lungcan.2011.10.021 22115704

[24] Palmirotta R Lovero D D'Oronzo S Todisco A Internò V Mele F. Pulmonary enteric adenocarcinoma: an overview. Expert Rev Mol Med. (2020) 22:e1. doi: 10.1017/erm.2020.2 32340641

[25] Gong J Fan Y Lu H. Pulmonary enteric adenocarcinoma. Trans Oncol. (2021) 14:101123. doi: 10.1016/j.tranon.2021.101123 PMC8141771 34000642

[26] Lin LI Xu CW Zhang BO Liu RR Ge FJ Zhao CH. Clinicopathological observation of primary lung enteric adenocarcinoma and its response to chemotherapy: A case report and review of the literature. Exp Ther Med. (2016) 11:201–7. doi: 10.3892/etm.2015.2864 PMC4726875 26889240

[27] Teranishi S Sugimoto C Nagayama H Segawa W Miyasaka A Hiro S. Combination of pembrolizumab with platinum-containing chemotherapy for pulmonary enteric adenocarcinoma. Cancer Diagnosis Prognosis. (2022) 2:253–7. doi: 10.21873/cdp.10102 PMC8962809 35399182

[28] Hu CH Shi S Dong W Xiao L Zang H Wu F. Hyperprogressive disease after immunotherapy: A case report of pulmonary enteric adenocarcinoma. Front Oncol. (2022) 12:799549. doi: 10.3389/fonc.2022.799549 35321429 PMC8937032

[29] Metro G Valtorta E Siggillino A Lauricella C Cenci M Ludovini V. Enteric-type adenocarcinoma of the lung harbouring a novel KRAS Q22K mutation with concomitant KRAS polysomy: a case report. ecancermedicalscience. (2015) 9:559. doi: 10.3332/ecancer.2015.559 26284123 PMC4531126

[30] Meng D Yuan M Li X Chen L Yang J Zhao X. Prognostic value of K-RAS mutations in patients with non-small cell lung cancer: a systematic review with meta-analysis. Lung Cancer. (2013) 81:1–10. doi: 10.1016/j.lungcan.2013.03.019 23608713

[31] Metro G Chiari R Bennati C Cenci M Ricciuti B Puma F. Clinical outcome with platinum-based chemotherapy in patients with advanced nonsquamous EGFR wild-type non-small-cell lung cancer segregated according to KRAS mutation status. Clin Lung Cancer. (2014) 15:86–92. doi: 10.1016/j.cllc.2013.08.002 24139827

